# Scoring Strategies Differentiating between Winning and Losing Teams during FIBA EuroBasket Women 2017

**DOI:** 10.3390/sports6020050

**Published:** 2018-05-29

**Authors:** Daniele Conte, Inga Lukonaitiene

**Affiliations:** Institute of Sport Science and Innovations, Lithuanian Sports University, 44221 Kaunas, Lithuania; inga.lukonaitiene@lsu.lt

**Keywords:** game-related statistics, performance analysis, basketball performance, team sports, basketball tactics

## Abstract

This study aimed to examine the scoring strategies differentiating between winning and losing teams during FIBA EuroBasket Women 2017 in relation to different game scores. Data were gathered for all games of FIBA EuroBasket Women 2017 from the official website. The investigated scoring strategies were fast break points (FBP); points in the paint (PP); points from turnover (PT); second chance points (SCP); and points from the bench (PB). Games were classified with cluster analysis based on their score difference as close, balanced, and unbalanced and the differences in the scoring strategies between winning and losing teams were assessed using magnitude-based statistics. Results revealed no substantial differences in FBP in any investigated cluster. Furthermore, winning teams showed a substantially higher number of PP and PT (in close and unbalanced games) and SCP (in balanced and unbalanced games) compared to losing teams. Finally, winning teams scored substantially lower and higher number of BPs in close games and unbalanced games, respectively, compared to losing teams. In conclusion, all the investigated scoring strategies discriminate between winning and losing teams in elite women’s basketball except for FBP. These results provide useful information for basketball coaches to optimize their training sessions and game strategies.

## 1. Introduction

Basketball is one of the most popular sports worldwide and in particular women’s basketball is increasing its popularity [1]. In the last few years, an increasing number of researchers have quantified the performance profile of women’s basketball from a physical and physiological standpoint, documenting that women’s basketball games are characterized by intermittent high-intensity efforts separated by short recovery periods and a high physiological demand [2,3]. In addition, the technical and tactical performance profile of women’s basketball games has been well investigated [4,5,6,7]. From a tactical standpoint, previous studies investigated the most effective tactical parameter during ball possessions, documenting that fast break might be one of the main indicators differentiating between winning and losing teams in both women and men’s basketball [8,9]. Indeed, it has been demonstrated that winning teams perform a higher number of fast break actions than losing teams [9]. Intuitively, performing more fast break actions would produce more scored points from this action. In addition, further studies documented that the use of the inside game might be considered a fundamental parameter in order to win a basketball game. In this regard, a previous investigation showed that ball possessions including the inside pass were the most effective [10]. However, no previous studies analyzed these indicators in women’s basketball. In addition, no studies verified whether the point scored with these tactical strategies (i.e., fast break and inside game actions) might be an indicator able to differentiate between winning and losing teams. In fact, both fast break and inside game strategies might correspond to a higher scored fast break points and points in the paint [10,11]. Therefore, further studies investigating these scoring strategies are warranted.

From a technical standpoint, many studies investigated the game-related statistics differentiating between winning and losing teams in women’s basketball [5,7,12]. Previous investigations identified that two of the game-related statistics most discriminating between winning and losing teams are turnovers and rebounds in women’s basketball [6,7,12]. Possibly, turnovers provide more opportunities for the opponents to score a basket since the opposing team might steal the ball and run fast break, outnumbering the defense [11]. Similarly, offensive rebounds create a second chance to score for the offensive teams. However, no previous studies investigated whether the points scored from turnover and the second chance points are performance indicators differentiating between winning and losing teams. Therefore, future studies should deeply investigate these aspects. In addition, the bench players’ performance can be considered as one of the possible determinants of a win in elite basketball [13]. Previous investigations indicated that bench players might provide a fundamental contribution to win a game, in particular for high-ranked teams [13,14]. Sampaio et al. [13] documented that starter players performed a higher number of defensive rebounds and assists. However, it has been demonstrated that the best teams possibly lose games because of the worse performance of bench players and particularly their offensive performance [13]. Indeed, bench players receive a statistically lower number of fouls and consequently score fewer points from free throws [13]. Therefore, the points scored by bench players might be a discriminant factor differentiating between winning and losing teams. Since it was not previously investigated whether points from the bench might discriminate between winning and losing teams, future studies should address this issue.

The above-mentioned scoring strategies might change in relation to different game scores. Indeed, games with a low or high score difference showed different performance indicators differentiating between winning and losing teams in elite women’s basketball [6]. Therefore, the aim of the study was to examine the scoring strategies differentiating between winning and losing teams during FIBA EuroBasket Women 2017 in relation to different game scores.

## 2. Materials and Methods

### 2.1. Subjects

The study was approved by an institutional review board, and meets the ethical standards in sports and exercise science research [15]. The game related statistics of all 40 games played in the FIBA EuroBasket Women 2017 were investigated (average score difference: 11.9 ± 8.6 points).

### 2.2. Procedures

In the tournament, sixteen teams competed in four groups at the preliminary round. Only the top two teams from each group qualified for the final stages (i.e., quarterfinals and final four) competing for the 1st–8th place. Data were gathered from the official box score on the website of the FIBA EuroBasket Women 2017 (http://www.fiba.basketball/eurobasketwomen/2017). The considered game-related statistics referring to scoring strategies were as follows: (a) fast break points (FBP), which refer to the points scored during fast break actions; (b) points in the paint (PP), which indicate the point scored in the key area; (c) points from turnover (PT), which refer to points scored after a turnover made by the opposite team; (d) second chance points (SCP), which refer to points scored after an offensive rebound; (e) points from the bench (PB), which refer to the amount of points scored by bench players.

### 2.3. Statistical Analysis

Games were classified based on their score difference through a hierarchical cluster analysis using Ward’s method and the Squared Euclidian distance as interval. The game classification through cluster analysis has been previously used in literature since it can provide more details on the relevance of the analyzed basketball games [16,17]. The hierarchical cluster analysis was performed using the software SPSS (Version 25.0). A magnitude-based statistics approach was applied to assess the chance of true differences (i.e., greater than the smallest worthwhile change) between winning and losing teams in each cluster for each performance indicator. All data were log-transformed for analysis to reduce bias arising from non-uniformity error and then analyzed for practical significance using magnitude-based inferences on a modified statistical spreadsheet [18]. Data were expressed as mean ± standard deviation, with pairwise comparisons determined using percentage of mean difference and effect size statistics (Cohen’s d) with 90% confidence intervals. The smallest worthwhile change was calculated as a standardized small effect size (0.2) multiplied by the between-subject standard deviation. Chances of real differences in variables were assessed qualitatively as: <1% = almost certainly not; 1–5% = very unlikely; 5–25% = unlikely; 25–75% = possibly; 75–95% = likely; 95–99% = very likely; and >99% = most likely. Clear effects greater than 75% were considered substantial [19]. If the chances of a variable having higher and lower differences were both >5%, the true effect was deemed to be unclear. Effect sizes were rated as follows: <0.20 = trivial; 0.20–0.59 = small; 0.60–1.19 = moderate; 1.20–1.99 = large; and >2.00 = very large [19].

## 3. Results

**Figure 1:**
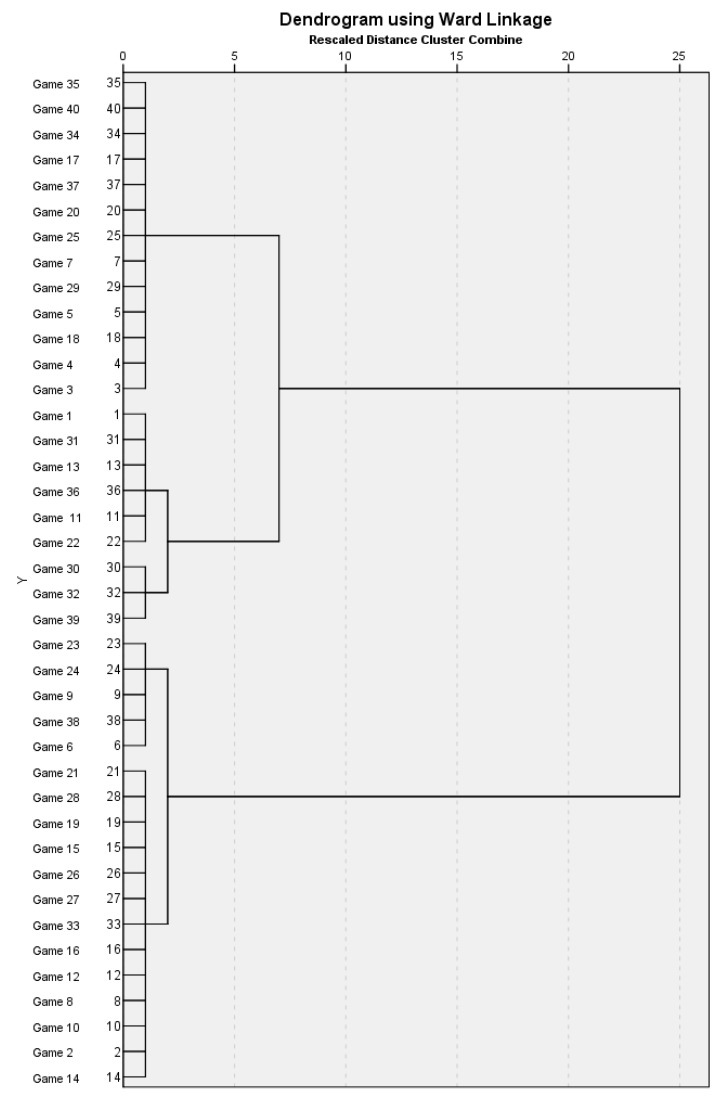
Dendrogram representing the three groups resulting from the hierarchical cluster analysis.

Cluster analysis grouped the analyzed games in 18 close, 13 balanced and 9 unbalanced games (score difference: 1–9 points; 10–19 points; 20–33 points, respectively) (Figure 1). The differences between winning and losing teams in each cluster for each performance indicator are shown in Table 1. In close games, winning teams showed a substantially higher number of points in the paints (likely negative) and points from turnover (likely negative), and a lower number of points from the bench (likely positive) compared to losing teams. No substantial differences (unclear) were shown for the other analyzed performance indicators. In balanced games, the only substantial difference found was for second chance points (likely negative). Considering unbalanced games, winning teams revealed a higher number of points in the paint (most likely negative), points from turnover (very likely negative), second chance points (very likely negative), and points from the bench (most likely negative) compared to losing teams.

## 4. Discussion

The aim of the study was to examine the scoring strategies differentiating between winning and losing teams during FIBA EuroBasket Women 2017 according to final score differences (close, balanced, and unbalanced games). Results revealed that (a) no substantial differences were shown in FBP in any investigated cluster; (b) winning teams showed a substantially higher number of PP and PT (in close and unbalanced games) and SCP (in balanced and unbalanced games) compared to losing teams; (c) winning teams scored substantially lower and higher number of BPs in close games and unbalanced games, respectively, compared to losing teams.

While the game-related statistics differentiating between winning and losing teams have been widely investigated in women’s basketball [5,6,7,12], little information is available on the scoring strategies adopted by these teams during games. Interestingly, an unclear difference was shown in FBPs scored between winning and losing teams. Previous studies demonstrated that the fast break is one of the most important offensive actions differentiating between winning and losing teams in elite men and women’s basketball [8,9,11,20]. Indeed, the fast break action is characterized by a high scoring percentage (i.e., 63–73%) since defense is usually outnumbered and/or not properly organized [11,21]. The unclear difference found between winning and losing teams in the FBPs scored indicates that fast break action is not one of the parameters differentiating between winning and losing teams in the EuroBasket Women 2017 championship. This finding might be explained by the tactical strategies adopted during EuroBasket Women 2017. Possibly, both winning and losing teams were performing fewer fast break and more set-offense actions in a tournament scenario like EuroBasket Women 2017, which is characterized by a congested match schedule compared to the national championship [22]. A previous investigation analyzing the tactical demand of tournament and seasonal games demonstrated 16% fewer fast break actions during tournament games and a longer mean duration of ball possessions [22]. The authors of this study suggested that this difference might be attributable to a higher level of the opponents with more developed defensive systems able to deny early scoring opportunities in international tournaments. Moreover, the fast break action requires a high level of physical fitness [11], while in a tournament scenario with a congested match schedule, players might have to slow down their pace to prevent possible fatigue toward the end of the competition [23]. Future investigations should assess whether the fast break action is a parameter discriminating between winning and losing teams in both women’s elite tournament and seasonal championships.

The results of our study also identified PP as one of the main indicators differentiating between winning and losing teams particularly in unbalanced and close games. This result might be explained by the possible importance of inside games in women’s basketball. The interaction between outside and inside players has been suggested to be a crucial element in European basketball and in NBA [10,24]. Indeed, Courel et al. [10] demonstrated an increase in the effectiveness of ball possessions including the inside pass from 49.8% to 63.3% in the Spanish professional male league. The inside game has been suggested to be fundamental in discriminating between winning and losing teams also in college basketball due to a substantially higher number of post entries (i.e., a pass from another position to the post area) documented by winning teams [25]. The importance of the inside games has been also documented in women’s basketball [26]. Gomez et al. [26] showed that the action completed in the key area reported the highest effectiveness in the women’s professional basketball league. Therefore, the results of our investigation possibly substantiate the importance of playing the inside game tactics in elite women’s basketball.

A further scoring strategy adopted substantially more by winning teams regards the PT. This result might be a consequence of the fact that losing teams performed more turnovers during the games. Indeed previous investigations analyzing the game-related statistics highlighted that turnover is the main parameter differentiating between winning and losing teams in women’s basketball [7,12]. Thus, our result confirms this idea that turnover possibly creates many scoring opportunities for the opponents teams. 

The analysis of SCP demonstrated that although winning teams scored a substantially higher number of points deriving from second chances in unbalanced and balanced games, an unclear difference was shown in close games. Previously, Gomez et al. [26] highlighted that elite women’s basketball teams obtained a higher offensive effectiveness when starting their attack in the offensive key area, probably due to offensive rebounds [26]. Conversely, a previous investigation analyzing the number of offensive rebounds in winning and losing college teams in close games documented an unclear difference [25]. Therefore, our findings possibly substantiate this result, highlighting that points scored from a second chance (i.e., mainly offensive rebounds) might be not a discriminant parameter between winning and losing teams in basketball close games. Considering these results, further studies should investigate this issue.

The analysis of PB highlighted contrasting results in unbalanced and close games. Winning teams scored a substantially higher number of PB compared to losing teams in unbalanced games, possibly due to the use of more bench players for winning teams during the garbage time likely to allow their best players to recover for the subsequent phases of the tournament. Conversely, winning teams showed a substantially lower number of points scored by bench players compared to losing teams in close games. A possible explanation for our result may be that losing teams were substituting more players, possibly to recover from the disadvantaged situation. This would allow more playing time for bench players, allowing them to score more. Indeed, playing time has been shown to be positively related to shooting performance in the male 1st division Spanish championship [14]. Therefore, our results possibly substantiate the importance of high-quality bench players, and call for future studies investigating their scoring effectiveness in relation to playing time in women’s basketball.

Although this study provides new information regarding the scoring strategies differentiating between winning and losing teams, it presents some limitations. Indeed, it only focused on the points scored but not effectiveness of the investigated actions. Moreover, the use of different statistical procedures might provide new insights regarding the association between the investigated scoring strategies and the possibility to win. Therefore, future studies should focus on investigating the effectiveness of the fast break, inside game, actions deriving from turnovers and offensive rebounds, and of substituting players using the notational analysis technique and further statistical approaches such as binary logistic regression or the conditional inference classification tree.

In conclusion, this study provides information on some of the most adopted scoring strategies differentiating between elite women’s winning and losing teams according to different game scores. Overall, FBP do not differentiate between winning and losing teams in each investigated cluster. All the other investigated scoring strategies differentiate between winning and losing teams in unbalanced and close games, except for SCP, which demonstrated an unclear difference in close games. These findings might provide useful information for basketball coaches to optimize their training sessions and game strategies.

## Figures and Tables

**Table 1:** Scoring strategies for winning and losing teams in relation to different game scores (close, balanced, and unbalanced games) expressed as mean ± standard deviation (SD), percentage (%), mean difference, and effect size (ES) with their 90% confidence intervals (CI) and magnitude-based inference.

Clusters	Scoring Strategies	Game Outcome		Losing vs. Winning Teams Comparisons		
		Winning Teams	Losing Teams	Mean Difference (90% CI)	ES (90% CI)	Magnitude-Based Inference
Close games	Fast break points	7.6 ± 4.4	6.8 ± 3.2	−0.7 (−2.9; 1.5)	−0.17 (−0.73; 0.39)	Unclear (14/40/47)
	Points in the paint	30.1 ± 6.2	26.3 ± 6.4	−3.8 (−7.3; −0.2)	−0.55 (−1.10; 0.00)	Likely negative (1/13/85)
	Points from turnover	12.8 ± 4.1	10.8 ± 3.7	−1.9 (−4.1; 0.3)	−0.43 (−0.98; 0.12)	Likely negative (3/21/76)
	Second chance points	7.2 ± 3.9	7.2 ± 3.6	−0.1 (−2.2; 2.0)	−0.10 (−0.66; 0.46)	Unclear (19/43/38)
	Points from the bench	13.1 ± 6.8	18.1 ± 9.9	5.1 (0.3; 9.8)	0.56 (0.01; 1.11)	Likely positive (86/12/1)
Balanced games	Fast break points	7.8 ± 3.0	6.5 ± 4.2	−1.2 (−3.7; 1.2)	−0.27 (−0.94; 0.40)	Unclear (12/31/57)
	Points in the paint	28.3 ± 7.7	27.1 ± 6.4	−1.2 (−6.0; 3.5)	−0.13 (−0.78; 0.52)	Unclear (19/37/43)
	Points from turnover	13.2 ± 5.3	12.5 ± 7.1	−0.8 (−5.0; 3.5)	−0.24 (−0.89; 0.41)	Unclear (13/33/54)
	Second chance points	10.0 ± 4.9	7.0 ± 2.3	−3.0 (−5.6; −0.4)	−0.53 (−1.18; 0.12)	Likely negative (3/16/80)
	Points from the bench	18.5 ± 9.1	21.6 ± 9.4	3.1 (−3.1; 9.3)	0.38 (−0.27; 1.04)	Unclear (68/25/7)
Unbalanced games	Fast break points	7.1 ± 5.3	5.3 ± 5.1	−1.8 (−6.1; 2.5)	−0.23 (−1.04; 0.58)	Unclear (18/29/53)
	Points in the paint	30.4 ± 5.5	19.1 ± 4.1	−11.3 (−15.4; −7.3)	−2.18 (−2.97; −1.39)	Most likely negative (0/0/100)
	Points from turnover	15.1 ± 4.8	8.6 ± 6.4	−6.6 (−11.3; −1.8)	−1.04 (−1.87; −0.21)	Very likely negative (1/4/95)
	Second chance points	9.3 ± 4.5	5.7 ± 2.0	−3.7 (−6.6; −0.7)	−1.06 (−1.85; −0.27)	Very likely negative (1/3/96)
	Points from the bench	30.3 ± 11.4	12.3 ± 8.9	−18.0 (−26.4; −9.6)	−1.58 (−2.36; −0.79)	Most likely negative (0/0/100)
